# Ubiquitination of CXCR7 Controls Receptor Trafficking

**DOI:** 10.1371/journal.pone.0034192

**Published:** 2012-03-23

**Authors:** Meritxell Canals, Danny J. Scholten, Sabrina de Munnik, Mitchell K. L. Han, Martine J. Smit, Rob Leurs

**Affiliations:** Leiden/Amsterdam Center for Drug Research, Division of Medicinal Chemistry, Faculty of Science, VU University Amsterdam, Amsterdam, The Netherlands; Chinese University of Hong Kong, Hong Kong

## Abstract

The chemokine receptor CXCR7 binds CXCL11 and CXCL12 with high affinity, chemokines that were previously thought to bind exclusively to CXCR4 and CXCR3, respectively. Expression of CXCR7 has been associated with cardiac development as well as with tumor growth and progression. Despite having all the canonical features of G protein-coupled receptors (GPCRs), the signalling pathways following CXCR7 activation remain controversial, since unlike typical chemokine receptors, CXCR7 fails to activate Gα_i_-proteins. CXCR7 has recently been shown to interact with β-arrestins and such interaction has been suggested to be responsible for G protein-independent signals through ERK-1/2 phosphorylation. Signal transduction by CXCR7 is controlled at the membrane by the process of GPCR trafficking. In the present study we investigated the regulatory processes triggered by CXCR7 activation as well as the molecular interactions that participate in such processes. We show that, CXCR7 internalizes and recycles back to the cell surface after agonist exposure, and that internalization is not only β-arrestin-mediated but also dependent on the Serine/Threonine residues at the C-terminus of the receptor. Furthermore we describe, for the first time, the constitutive ubiquitination of CXCR7. Such ubiquitination is a key modification responsible for the correct trafficking of CXCR7 from and to the plasma membrane. Moreover, we found that CXCR7 is reversibly de-ubiquitinated upon treatment with CXCL12. Finally, we have also identified the Lysine residues at the C-terminus of CXCR7 to be essential for receptor cell surface delivery. Together these data demonstrate the differential regulation of CXCR7 compared to the related CXCR3 and CXCR4 receptors, and highlight the importance of understanding the molecular determinants responsible for this process.

## Introduction

CXCL12 (SDF1α)-mediated effects have been classically attributed to its interaction with chemokine receptor CXCR4. However, it has recently been appreciated that CXCL12 also binds with high affinity to chemokine receptor CXCR7 (earlier also referred to as RDC-1 or CXC-CKR2), an evolutionary conserved G protein-coupled receptor (GPCR) [1], [2]. In addition, the CXCR3-ligand CXCL11 (I-TAC) [1], [2] has also been found to bind to CXCR7. CXCR7 plays a role in cardiac development [3] as well as in promoting tumor development and progression [4], [5]. In fact, CXCR7 has been shown to promote the growth of tumors formed from lung, breast and liver cancer cells [4], [6] and increased expression of CXCR7 has been correlated with the aggressiveness of prostate cancer [7], suggesting an important role for this receptor in tumor metastases and progression [8]. More recently, it has been shown that CXCR7 is also expressed in the nervous system, where it has been described to be involved in both the development of the CNS [9], [10] as well as in tumor malignancy [11]. Importantly, in cortical interneurons, CXCR7 has been postulated to indirectly regulate the expression of CXCR4 and consequently sustain normal levels of this receptor [12]. Similarly, in zebrafish, CXCR7 is critical for the proper migration of primordial germ cells [13]. Such an emerging role for CXCR7 in both normal development and cancer are motivating ongoing efforts to target this receptor therapeutically. However, molecular interactions and signaling events following CXCL11 or CXCL12 binding to CXCR7 remain poorly defined and controversial.

Several reports suggest that CXCR7, despite conserving most of the canonical GPCR features, does not activate Gα_i_-mediated pathways that are typical for chemokine receptors and would result in GTP hydrolysis, calcium mobilization, and chemotaxis [2], [3], [14]. In contrast, other studies suggest CXCR7 as a modulator of CXCR4-mediated signaling through CXCR7-CXCR4 heterodimerization. Indeed, the presence of CXCR7 has a dramatic effect on the signaling derived from CXCR4 activation [14]–[16]. Another hypothesis on the physiological function of CXCR7 suggests its role as a “decoy” receptor or chemokine scavenger. Internalization upon binding of CXCL11 or CXCL12 would generate the gradient of chemokine necessary for a correct CXCR4 migratory response [12], [13], [17], [18], without any signaling following chemokine binding to CXCR7. Yet, some of these decoy receptors have been shown to be constitutively internalized by a β-arrestin-mediated mechanism [19]. It has recently been described that CXCR7 also interacts with β-arrestin in a ligand-dependent manner [15], [20], [21] and, more importantly, that this interaction results in ERK1/2 phosphorylation and translocation via a G protein-independent, β-arrestin-mediated signal [22], [23], suggesting different functions other than the “decoy” activity of this receptor.

As for all membrane proteins, the magnitude of the cellular response elicited by a ligand binding to a GPCR is dictated by the level of receptor expression at the plasma membrane, which is the balance of finely tuned endocytic and recycling pathways. Recent data reveal that receptor trafficking can have differential effects on the strength of the intracellular signaling cascade [24]. One of the most common events for receptor desensitization and internalization involves the recruitment of the β-arrestin protein, which binds to the activated and phosphorylated receptor. This uncouples the receptor from its G protein and scaffolds the binding of proteins involved in formation of clathrin-coated pits and receptor endocytosis [25]. Once internalized in early endosomes, some GPCRs are dephosphorylated and subsequently recycled back to the plasma membrane where they can again respond to agonists, a process termed resensitization. Alternatively, a subclass of GPCRs enter the degradation pathway, where they are targeted to lysosomes for proteolysis, giving rise to long-term attenuation of signaling or downregulation [25]. Despite recent advances, the mechanisms mediating endosomal sorting remain elusive; however, receptor ubiquitination, i.e. the covalent addition of the small protein ubiquitin to the lysine side chains of the substrate protein, has recently been reported to play an important role for several GPCRs. Recent studies suggest that mammalian GPCR ubiquitination is essential for lysosomal sorting but not for receptor internalization [26], [27]. Direct β_2_-adrenergic receptor (β_2_AR) ubiquitination is not required for internalization but regulates lysosomal sorting and degradation of activated receptors [27]. Similar to β_2_AR, ubiquitination of CXCR4 is essential for agonist-promoted receptor lysosomal degradation but not for internalization [26]. In contrast, the protease-activated receptor-1 (PAR-1) has been shown to be basally ubiquitinated and de-ubiquitinated after receptor activation, revealing a novel function for ubiquitination in the regulation of GPCR internalization [28]. Several recent reports show that CXCR7 internalizes upon agonist stimulation and, recycles to the cell surface [17], [20], [21], [29]. However, the mechanisms involved in such regulation have not yet been identified. Thus, there is a need to define fundamental mechanisms for the activation and regulation of this receptor. In the present study we have investigated the molecular determinants responsible for CXCR7/β-arrestin interaction and CXCR7 regulation. We have identified C-terminal CXCR7 residues that are of key importance for its internalization and subsequent recycling. Importantly, and in contrast to what has been described for the closely related receptor CXCR4, we show that CXCR7 is basally ubiquitinated and investigate the role of ubiquitination/de-ubiquitination in CXCR7 regulation.

## Results

### CXCR7 C-terminal Serine/Threonine residues are essential for β-arrestin recruitment

**Figure 1:**
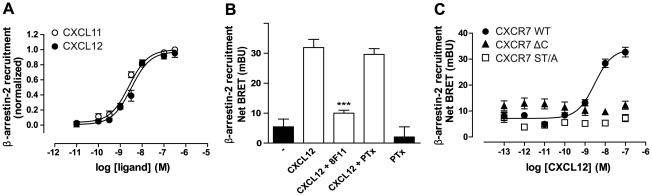
β-arrestin2 recruitment to CXCR7 is dependent on C-terminal Ser/Thr residues. (**A**) CXCL11 or CXCL12-mediated β-arrestin2 recruitment to CXCR7. HEK293T co-expressing RLuc-tagged CXCR7 and YFP-tagged β-arrestin2 were stimulated with increasing concentrations of CXCL11 (open circles) or CXCL12 (filled circles) (**B**) HEK293T co-expressing RLuc-tagged CXCR7 and YFP-tagged β-arrestin2 were incubated overnight with 25 ng/ml of PTX or for 30 min with the CXCR7-specific antibody 8F11 prior to the BRET measurement. (**C**) CXCL12-induced β-arrestin2 recruitment to CXCR7 wt (filled circles), a truncated CXCR7 lacking the C-terminus (CXCR7 ΔC, filled triangles) or a mutant CXCR7 for which all the Ser and Thr residues were mutated to Ala (CXCR7 ST/A, open squares). HEK293T cells coexpressing *RLuc*-tagged CXCR7 mutants and YFP-tagged β-arrestin2 were stimulated with increasing concentrations of CXCL12 prior to BRET measurements. Data represent the mean ± SEM of 4 experiments each performed in triplicate. Results are expressed in Net BRET as described in Materials and Methods. ***, p<0.001 by one-way ANOVA and Bonferroni post test.

In order to investigate potential CXCR7-mediated signaling, we evaluated CXCR7 activation in several assays that have been traditionally linked to chemokine receptor activation. Unlike classical chemokine receptors, and despite being expressed at the cell surface, CXCR7 does not show Gα_i_ protein coupling as assessed by [^35^S]GTPγS accumulation assay or inhibition of forskolin-induced cAMP levels (**Fig. S1** and [14]). Additionally, no CXCR7-mediated response was observed in cAMP and inositol phosphates accumulation experiments, ruling out the possibility of any detectable Gα_s_ and Gα_q/11_ protein activation (data not shown). More recently, the ability of CXCR7 to recruit β-arrestin has emerged as the potential initial signaling step after receptor activation [15], [20], [21], [23]. In agreement with this, we detected β-arrestin recruitment to CXCR7 using a BRET approach. In HEK293T cells transiently transfected with CXCR7-RLuc and β-arrestin2-YFP, a dose-dependent increase in energy transfer was observed when stimulating the receptor with CXCL11 or CXCL12 (pEC_50_ = 8.5±0.1 and 8.6±0.1 respectively) (**Fig. 1A**). Furthermore, β-arrestin1 recruitment to CXCR7 by CXCL11 and CXCL12 was induced with similar potencies as observed for β-arrestin2 (pEC_50_ = 8.7±0.1 and 8.6±0.1 for CXCL11 and CXCL12 respectively). The anti-CXCR7 antibody 8F11 blocked the chemokine-mediated β-arrestin recruitment in the transfected HEK293T cells, indicating that it is a CXCR7-specific effect (**Fig. 1B** and [21]). In addition, β-arrestin recruitment was shown to be Gα_i/o_ protein-independent, as an overnight treatment of the cells with pertussis toxin (PTX) had no effect on the ability of CXCR7 to interact with the scaffolding protein (**Fig. 1B**).

It has been shown for several GPCRs that β-arrestin binds to phosphorylated amino acid residues of the C-terminus of the GPCR protein [30]. We therefore generated a C-terminally truncated CXCR7 mutant, which lacks the 39 C-terminal amino acids after the NPXXY motif (CXCR7 ΔC, **Table S1**). Radioligand binding assays showed that the truncated receptor retained the same binding affinity for CXCL12 as the wild type (pK_d_ ± SEM, 9.7±0.1 and 9.3±0.1 for CXCR7 wt and CXCR7 ΔC, respectively, see **Table S2**). β-arrestin recruitment experiments using an RLuc-tagged form of CXCR7 ΔC confirmed that this truncated receptor was unable to recruit β-arrestin2 (**Fig. 1C**). In addition, mutation of all the serine (Ser) and threonine (Thr) residues in the C-terminus to alanine (Ala) residues (CXCR7 ST/A, **Table S1**) resulted in similar observations. CXCR7 ST/A was not able to recruit β-arrestin2 (**Fig. 1C**). As expected, CXCL12 displayed similar affinity for the CXCR7 ST/A mutant compared to the CXCR7 WT (pK_d_±SEM, 10.6±0.3, and 9.7±0.1, respectively, Table S2). Cell surface expression of RLuc-tagged CXCR7 ΔC or ST/A was confirmed by whole cell [^125^I]-CXCL12 binding, ruling out the possibility that the lack of β-arrestin recruitment is caused by the absence of the receptor mutants at the cell surface (**Fig. S2**).

### CXCR7 internalizes via clathrin-coated pits in a β-arrestin-dependent manner

**Figure 2:**
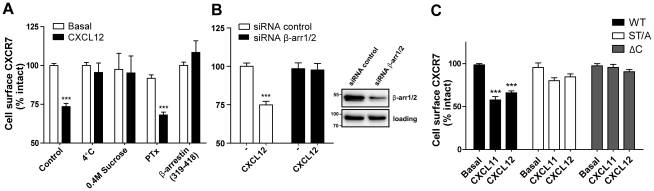
(A) CXCR7 internalization depends on CCPs and is G protein-independent. HEK293T cells were transfected with wt CXCR7 (and β-arrestin (319–418) were indicated) and cell surface levels of the receptor after CXCL12 stimulation was detected by ELISA using the CXCR7-specific antibody 11G8. Incubation with 0.4 M Sucrose was done 30 min prior and during stimulation. PTX was incubated overnight at 25 ng/ml final concentration. **(B) β-arrestin1/2 knock-down prevents CXCR7 internalization.** HEK293/CXCR7 cells transfected with control siRNAs (white bars) or pools targeting β-arrestin1/2 (filled bars), were stimulated with CXCL12 (10^−8^ M) or vehicle for 45 min and receptor surface expression was determined. Knockdown of β-arrestin1 and -2, 48 hrs after transfection, was assessed in western blot using an anti-β–arrestin1/2 antibody (inset). Anti-STAT3 (mAb 79D7, Cell Signaling Technologies) was used as loading control. **(C) CXCR7 C-terminus is essential for receptor internalization.** HEK293T cells were transfected with wt CXCR7 (filled bars), CXCR7 ΔC (grey bars) or CXCR7 ST/A (white bars) and cell surface receptor levels were assessed as above. Data represent the mean ± SEM of at least 3 experiments each performed in triplicate. ***, p<0.001 by one-way ANOVA and Bonferroni post test.

β-arrestins have classically been involved in GPCR signal termination and internalization by binding to activated/phosphorylated receptors. We therefore investigated CXCR7 regulation after agonist exposure (**Fig. 2**). After 45 min incubation with 10^−8^ M CXCL12, a decrease in cell surface CXCR7 expression was observed by ELISA using the specific CXCR7 antibody 11G8. This effect was mimicked by CXCL11 and was not affected by overnight PTX treatment (**Fig. 2A and 2C**). Internalization was blocked when the incubation was performed at 4°C or in presence of 0.4 M sucrose, typical inhibitors of receptor internalization [31] (**Fig. 2A**). Co-transfection of CXCR7 with the β-arrestin (319–418) dominant negative (DN), effectively inhibited agonist-induced CXCR7 internalization. β-arrestin (319–418) encodes for the last 100 aa of the C-tail of β-arrestin1 and effectively binds clathrin, but completely lacks the capacity to bind GPCRs. Consequently, overexpression of β-arrestin (319–418) DN depletes the clathrin-mediated endocytic machinery [32]. Therefore, these results suggest that CXCR7 rapidly internalizes upon CXCL11 and CXCL12 exposure by a mechanism dependent on clathrin-coated pits (CCPs). This result, together with the fact that the endocytic processing of CXCR7 is G protein-independent, suggests the involvement of β-arrestins in CXCR7 internalization. This hypothesis was further validated by performing internalization ELISA experiments in the presence of siRNA targeting β-arrestin1 and 2 (Fig. 2B). Transfection of HEK293/CXCR7 cells with a non-targeting siRNA pool had no effect on the previously observed CXCL12-induced internalization. However, transfection of siRNA pools targeting β-arrestin1 and -2, resulted in a significant knock-down of the endogenous levels of these proteins (as shown by Western blot analysis in **Fig. 2B**) and was able to completely inhibit CXCL12-mediated internalization of CXCR7 (**Fig. 2B**).

Furthermore, and in agreement with the previous results indicating the lack of β-arrestin recruitment, no internalization was observed after CXCL12 or CXCL11-stimulation of the CXCR7 ΔC or the CXCR7 ST/A mutant receptors since the cell surface receptor levels detected by ELISA were not significantly different in the presence or absence of either CXCL11 or CXCL12 (**Fig. 2C**). Altogether, these results demonstrate that CXCR7 internalization is mainly (if not only) β-arrestin-dependent and relies on the presence of the Ser and Thr residues in the C-terminus of the receptor.

### CXCR7 recycles to the cell surface after internalization

**Figure 3:**
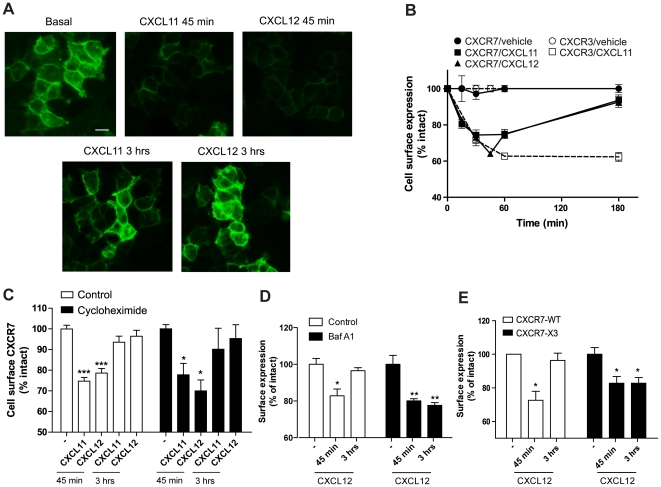
CXCR7 recycles to the cell surface after internalization. (**A**) HEK293T stably expressing CXCR7 were stimulated with 10^−8^ M CXCL11, CXCL12 or vehicle for 45 min or 3 h and fixed immediately. CXCR7 was detected using the specific 11G8 antibody and an Alexa-488-conjugated secondary antibody. Scale bar represents 10 µm. (**B**) HEK293T cells expressing CXCR7 (filled symbols) or CXCR3 (open symbols) were incubated with CXCL11 (10^−8^ M, squares), CXCL12 (10^−8^ M, triangles) or vehicle (circles) for the indicated times. Cell surface receptor levels were detected by ELISA using CXCR7- or CXCR3-specific antibodies (11G8 and mAB160, respectively). Results were normalized to basal surface protein levels, and data represent the mean ± SEM of 4 experiments each performed in triplicate. (**C**) ELISA was performed as in B in cells pre-incubated for 2 h with the *de novo* protein synthesis inhibitor cycloheximide (10 µg/ml). (**D**) ELISA performed as in C on intact HEK293/CXCR7 cells treated with vehicle or 1 µM of bafilomycin A1 (Baf A1), 30 min prior to incubation with CXCL12. **(E) C-terminal Ser/Thr clusters determine receptor fate after internalization.** HEK293T cells were transiently transfected with CXCR7 wt (white bars) or with a chimeric receptor consisting of CXCR7 harboring the C-terminal sequence of CXCR3 (CXCR7-X3, filled bars). To assess the cell surface expression of the receptor, ELISA experiments were performed after 30 min or 3 hours of incubation with 10^−8^ M CXCL12. Data represent the mean ± SEM of 3 experiments each performed in triplicate. ***, p<0.001, **, p<0.01, and *, p<0.05 by one-way ANOVA and Bonferroni post test.

Once internalized, GPCRs are either directed to late endosomes and processed for lysosomal degradation, or recycled to the cell surface [25]. To investigate the fate of internalized CXCR7 receptors, we stimulated HEK293 cells, stably expressing CXCR7, with 10^−8^ M of CXCL11 or CXCL12 and incubated them for 45 min or 3 hours and subsequently stained for CXCR7 immunoreactivity. In basal conditions, CXCR7 was uniformly distributed at the cell surface (**Fig. 3A**). However, after 45 min of chemokine incubation a marked decrease in cell surface staining was observed, in agreement with the results from the ELISA experiments and suggesting receptor internalization (**Fig. 2**). In contrast, after 3 hours incubation with chemokine, the level of cell surface CXCR7 expression was similar to the expression in basal conditions, indicating the reappearance of CXCR7 proteins at the cell surface (**Fig. 3A**). These results were then confirmed by time-course ELISA experiments. In cells transiently transfected with CXCR7, we observed receptor internalization after 1 hour of ligand stimulation and recovery of cell surface CXCR7 levels at 3 hours of chemokine incubation (**Fig. 3B**). In contrast, stimulation of CXCR3 with its agonist CXCL11, led to significant internalization after 1 hour, but no recycling to the cell surface was detected at later time points, indicating the occurrence of receptor downregulation (**Fig. 3B**). In addition, permeabilization of the cells showed a decrease of CXCR3 but not CXCR7 total receptor levels (**Fig. S3**) further highlighting the differences in post-endocytic sorting of both receptors. To rule out the detection of newly synthesized receptors after 3 hours of agonist incubation, cells were treated with the *de novo* protein synthesis inhibitor cycloheximide (10 µg/ml). This had no effect on the recovery of CXCR7 cell surface levels, suggesting that after internalization, CXCR7 recycles back to the cell surface, despite the continuous presence of chemokines (**Fig. 3C**). Several reports have recently suggested that CXCR7 can reside within intracellular pools [13], [17], [29] and therefore, mobilization of CXCR7 from such intracellular stores could also account for the reappearance of the receptor at the plasma membrane, even in presence of cycloheximide. To exclude this possibility we examined the effects of bafilomycin A1, an inhibitor of vacuolar-type H^+^-ATPases, on CXCR7 recycling. It has previously been shown that endosomal acidification promotes dissociation of ligand from GPCRs, and that inhibitors of such acidification prevent receptor recycling and resensitization [33], [34]. As shown in **Fig. 3D**, incubation of CXCR7 expressing cells with 1 µM bafilomycin A1 did not affect CXCR7 internalization after 45 min of CXCL12 stimulation. In contrast, bafilomycin A1 treatment did severely affect the reappearance of CXCR7 at the cell surface after 3 hours (**Fig. 3D**). These results therefore indicate that CXCR7 proteins are able to recycle to the plasma membrane after CXCL12 stimulation.

The presence of Ser and Thr clusters within the C-terminus of GPCRs is indicative of a stable interaction with β-arrestin and a slow or no recycling to the cell surface. Conversely, absence of such clusters induces a more transient interaction with β-arrestin and allows rapid recycling of the receptor to the cell surface [35]. Indeed, the sequence of the C-terminal tails of CXCR3 and CXCR7 differs substantially (**Table S1**). Whereas the C-terminus of CXCR3 contains Ser/Thr clusters, such a motif is not present in the CXCR7 C-terminus. To test the role of the C-terminus in receptor recycling, we generated a chimeric CXCR7 receptor harboring the C-terminal sequence of CXCR3 (CXCR7-X3). This chimeric receptor showed the same affinity for CXCL12 as the wild type receptor (9.7±0.1 vs 9.5±0.1, pK_d_ ± SEM, **Table S2**). ELISA experiments with transiently transfected HEK293T cells showed no recycling of this chimeric receptor after long-term agonist exposure, indicating that the presence of Ser/Thr clusters on the CXCR3 C-terminus determines its downregulation, whereas the absence of these clusters on the CXCR7 C-terminus, allows its re-routing to the cell surface (**Fig. 3E**). Unfortunately, generation of a chimeric receptor of CXCR3 with the C-terminus of CXCR7 (CXCR3-X7) resulted in a receptor with very limited cell surface expression whilst the total expression level was similar to that of the wild type CXCR3. These observations suggest the presence of molecular determinants on the CXCR3 C-tail sequence that are absent in CXCR7 and that are important for proper cell surface delivery of CXCR3. This observation is in line with previous results on the CXCR3 receptor where several C-terminal truncations result in poorly expressed receptors (Scholten et al., unpublished observations).

### CXCR7 is constitutively ubiquitinated and CXCL12 stimulation induces receptor de-ubiquitination

**Figure 4:**
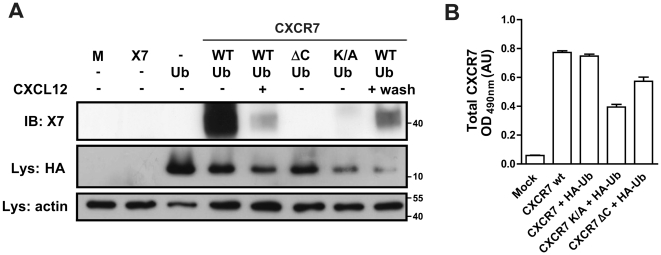
The C-terminus of CXCR7 is constitutively ubiquitinated. **(A) CXCR7 gets deubiquitinated by CXCL12-stimulation.** HEK293T cells were transfected as indicated and processed for immunoprecipitation of the HA-Ub (See Materials and Methods). (**A**) CXCR7 was stimulated with 10^−8^ M CXCL12 for 30 min, and removal of CXCL12 was performed by two washes of the cells and additional 30 min incubation with fresh chemokine-free media. Detection of the immunoprecipitated CXCR7 was done with the 11G8 antibody. HA-Ub expression was confirmed by blotting lysates using an anti-HA antibody and equal loading was controlled by detection of actin on the same blot. Molecular weight markers (kDa) are indicated on the right of the blot. (**B**) Detection of total CXCR7 protein expression by ELISA in the same cells.

Recently, it has been proposed that ubiquitination plays an important role in GPCR regulation [36]. Receptors such as the β_2_-adrenergic receptor, the vasopressin receptor, PAR-1 receptor and CXCR4 are all regulated by the covalent linkage of intracellular lysine residues and the small protein/peptide ubiquitin [26]–[28], [37]. Ubiquitination of CXCR4 by the E3 ubiquitin ligase AIP4 following activation with CXCL12 results in receptor downregulation [26]. Sequence comparison of the C-terminus of CXCR4 and CXCR7 revealed that, similar to CXCR4, CXCR7 contains several lysine (Lys) residues in its C-terminus. We therefore investigated the ubiquitination status of CXCR7 by co-immunoprecipitation experiments using HA-tagged ubiquitin (HA-Ub) and CXCR7 wt (**Fig. 4**). Interestingly, and in contrast to CXCR4, we observed that CXCR7 is ubiquitinated under basal conditions. In the lane corresponding to non-stimulated cells, a clear band could be detected after immunoprecipitation of HA-Ub and subsequent detection of CXCR7 with the 11G8 antibody (**Fig. 4**). This band was not observed when the co-immunoprecipitation was performed in samples expressing the CXCR7 ΔC or a CXCR7 mutant in which all Lys residues had been replaced by Ala (CXCR7 K/A), confirming ubiquitination of CXCR7 at C-terminal Lys residues. Moreover, activation of CXCR7 by CXCL12 induced a rapid receptor de-ubiquitination, as the intensity of the band corresponding to ubiquitinated receptor decreased significantly after 30 min incubation with CXCL12 (10^−8^ M). Conversely, removal of the chemokine by subsequent washout steps resulted in the reappearance of the band corresponding to the ubiquitinated state of CXCR7 (**Fig. 4**) suggesting that ligand-induced de-ubiquitination of CXCR7 is a reversible process.

**Figure 5:**
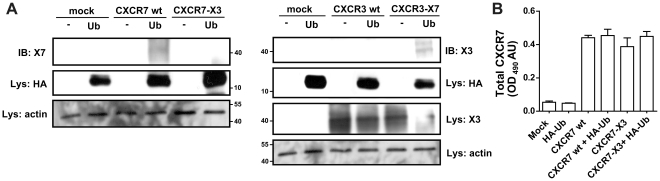
CXCR7/CXCR3 tail switch alters ubiquitination properties of the receptors. (**A**) Immunoprecipitation experiments were performed in cells expressing chimeric receptors consisting on CXCR7 with CXCR3 C-terminus (CXCR7-X3) or the reciprocal CXCR3 with CXCR7 C-terminus (CXCR3-X7). Detection of the immunoprecipitated CXCR7 and CXCR3 was done with the 11G8 and mAB160 antibodies, respectively. HA-Ub expression was confirmed blotting lysates using an anti-HA antibody and equal loading was controlled by detection of actin on the same blot. Molecular weight markers (kDa) are indicated on the sides of the blots. (**B**) Detection of total CXCR7 protein expression by ELISA in the same cells.

In contrast to CXCR7, the chemokine receptor CXCR3 has no Lys residues in its C-terminus. We therefore performed similar co-immunoprecipitation experiments in cells transfected with the chimeric receptor CXCR7-X3 and the reciprocal CXCR3-X7. As shown in **Fig. 5**, and in agreement with the results obtained with the CXCR7 ΔC and CXCR7 K/A mutants, the introduction of the CXCR3 tail sequence on the CXCR7 tail resulted in a receptor unable to undergo basal ubiquitination, whereas introducing Lys residues in the CXCR3 receptor resulted in a mutant receptor able to be co-immunoprecipitated with HA-Ub.

**Figure 6:**
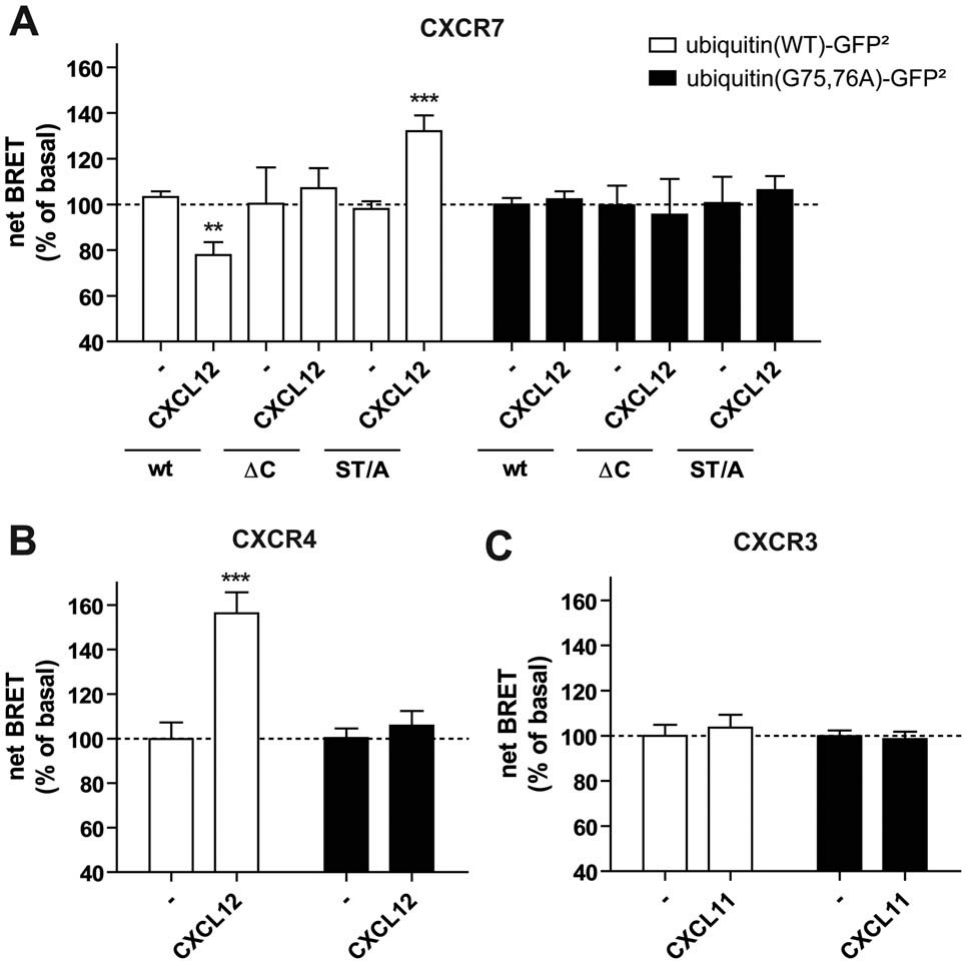
Real-time monitoring of receptor ubiquitination using BRET^2^. HEK293T cells were transfected with Ub-GFP^2^ (white bars) or (G75A,G76A)-Ub-GFP^2^ (filled bars) and (**A**) CXCR7-RLuc, CXCR7 ΔC-RLuc, or CXCR7 ST/A-Rluc, (**B**) CXCR4-RLuc, or (**C**) CXCR3-RLuc. BRET^2^ was measured 30 min after stimulation with 10^−8^ M of CXCL12 (CXCL11 for CXCR3) by addition of coelenterazine 400a and immediate read out. Results are expressed in Net BRET normalized to basal as described in Materials and Methods. Data represent the mean ± SEM of 3 experiments each performed in triplicate. **, p<0.01, and ***, p<0.001, by Student t test.

To confirm the constitutive ubiquitination of CXCR7 and the subsequent de-ubiquitination after CXCL12 activation, we performed BRET^2^ experiments that allow real-time monitoring of receptor ubiquitination [38]. In these experiments, cells were co-transfected with RLuc-tagged CXCR7 and a GFP^2^-tagged ubiquitin or, as a negative control, a (G75A, G76A)-Ub-GFP^2^ mutant that is unable to take part in the ubiquitination process [38]. In cells transfected with CXCR7-RLuc as energy donor, a decrease of the BRET response was detected after 30 min incubation with 10^−8^ M CXCL12. These data are in agreement with the suggested CXCR7 de-ubiquitination after agonist exposure (**Fig. 6**). In addition, no change in energy transfer was observed when the (G75A, G76A)-Ub-GFP^2^ mutant was co-expressed with CXCR7-RLuc (**Fig. 6A**). When the same experiment was performed with the CXCR7 ΔC-RLuc (**Fig. 6A**) or CXCR3-RLuc (**Fig. 6C**) as energy donors, agonist stimulation did not induce any change in BRET^2^ indicating the absence of modulation of the ubiquitination state of these receptors. Interestingly, when the phosphorylation-deficient mutant CXCR7 ST/A-RLuc was used, we observed an increase of receptor ubiquitination upon CXCL12 stimulation. This result suggests that, in the absence of β-arrestin recruitment, CXCR7 cannot undergo de-ubiquitination and, most importantly, that the ubiquitin-conjugated CXCR7 is the prevalent form at the cell surface. Finally, when CXCR4-RLuc was used as energy donor, CXCL12 stimulation induced an increase in energy transfer (**Fig. 6B**), which is in agreement with previous reports on increased CXCR4 ubiquitination after agonist exposure and therefore suggesting differential regulation of the two CXCL12 receptors [26].

### C-terminal lysine residues are important for correct CXCR7 trafficking to the cell surface

**Figure 7:**
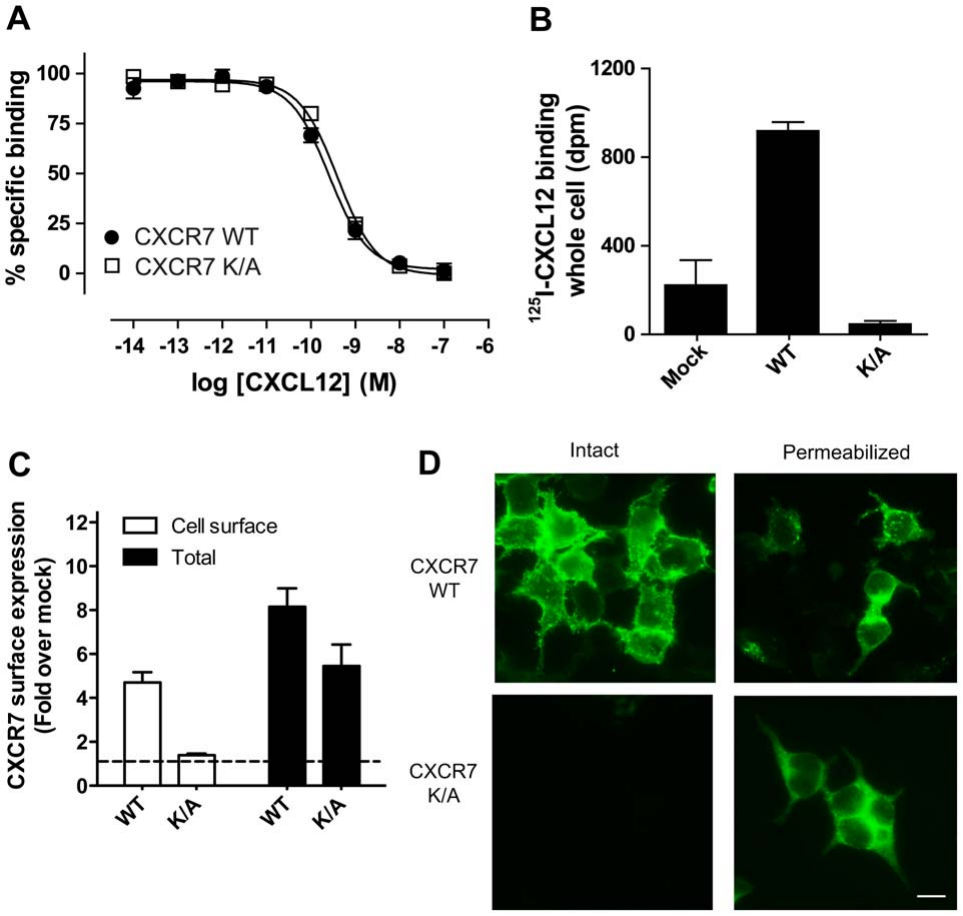
CXCR7 C-terminal Lys residues are important for correct trafficking of the receptor to the cell surface. (**A**) [^125^I]CXCL12 radioligand binding in total membranes of HEK293T cells transfected with CXCR7 wt (filled circles) or CXCR7 K/A (open squares) shows that both receptors display the same affinity for CXCL12 (pK_d_ = 9.7±0.1 and 9.5±0.1 respectively). (**B**) [^125^I]CXCL12 radioligand binding was performed in whole cells expressing pcDEF_3_ (mock) CXCR7 wt (WT) or CXCR7 K/A (K/A) (**C**) CXCR7 wt or CXCR7 K/A expressing cells were fixed (open bars) or fixed and permeabilized (filled bars) and CXCR7 was detected by ELISA using CXCR7-specific antibody 11G8. (**D**) Immunocytochemistry of HEK293T cells transfected with CXCR7 wt (upper panels) or CXCR7 K/A (lower panels). Cells were fixed (left panels) or fixed and permeabilized (right panels) and detection of CXCR7 was performed with 11G8 antibody. Scale bar represents 10 µm.

Despite normal binding characteristics when assessed by [^125^I]-CXCL12 membrane binding, no cell surface expression could be detected for the CXCR7 K/A mutant when performing [^125^I]-CXCL12 whole cell binding, intact cell ELISA and intact cell immunocytochemistry (**Fig. 7**). However, the CXCR7 K/A mutant could be detected after permeabilization of cells in both, ELISA and immunocytochemistry experiments (**Fig. 7C and D**). Under these conditions CXCR7 WT was distributed in punctate intracellular vesicles whereas the K/A mutant was uniformly distributed in the cytoplasm and displaying a marked colocalization with β-arrestin (**Fig. S4**). Such colocalization is in agreement with the fact that, although being unable to detect an CXCL12-dependent β-arrestin recruitment for CXCR7 K/A (due to its absence from the cell surface), we could observe an increased receptor- β-arrestin2 BRET signal in the basal state of CXCR7 K/A when compared to the wild type receptor (**Fig. S5**). These results suggest that although its ability to bind chemokines remains unaltered, the absence of C-terminal Lys residues results in constitutive internalization and intracellular retention of CXCR7, demonstrating the importance of these residues in the C-terminus of CXCR7 for the correct trafficking of this receptor to the cell surface.

## Discussion

Due to its potential role in cancer development and progression, the recently deorphanized chemokine receptor CXCR7 has become a potential therapeutic target for the treatment of a variety of tumors [4], [7], [8]. In addition, the fact that CXCR7 binds CXCL12 with high affinity requires a revisit of some observed effects thought to be solely mediated by CXCR4 and a detailed understanding of the biochemistry and pharmacology of CXCR7. An initial step in the characterization of CXCR7 is to establish the mechanisms that regulate its expression at the cell surface. The regulatory processes for CXCR4 have been extensively studied and it has been demonstrated that CXCR4 is phosphorylated by several kinases at specific sites that result in differential CXCL12-induced signaling [39]. β-arrestin recruitment to phosphorylated residues of the CXCR4 C-terminus has been shown to mediate not only receptor internalization but also CXCL12-mediated chemotaxis via p38 MAPK [40]–[42]. In addition, it has also been demonstrated that the interaction between β-arrestin and CXCR4 targets the receptor for lysosomal degradation [43]. In the present report we show that β-arrestin recruitment is also the main component of the endocytic machinery that internalizes CXCR7 after agonist exposure. Interestingly, despite the difference in CXCR7 affinities of CXCL11 and CXCL12 in radioligand binding studies, they have similar potencies in β-arrestin recruitment. We have identified the Ser/Thr residues of the C-tail of the receptor to be essential for β-arrestin interaction as well as CXCR7 internalization, given the findings that CXCR7 ΔC and CXCR7 ST/A fail not only to recruit β-arrestin but also to internalize. As intracellular Ser and Thr residues represent the phosphorylation sites of GPCRs that lead to arrestin recruitment and subsequent desensitization and internalization, our results suggest the existence of one or several kinases responsible of CXCR7 phosphorylation. Further investigation is required to determine the nature of this phosphorylation and which kinases (e.g. GRKs) are involved. Both β-arrestin recruitment and receptor internalization are G protein-independent as shown by their PTX insensitivity. In addition, a clathrin-coated pit endocytic mechanism could also be suggested from the inhibition of CXCR7 internalization by the β-arrestin (319–418) peptide. Finally, the ability of β-arrestin1/2 siRNA to completely block receptor internalization provided further evidence for the involvement of these proteins in the regulation of CXCR7.

A sequence comparison of the C-terminal tail of CXCR7 and CXCR3 highlighted the presence of Ser/Thr clusters in the latter receptor. Such Ser/Thr clusters have been proposed to be responsible for a strong interaction with β-arrestin thus inducing slow internalization and, eventually, receptor downregulation [35]. This was in fact observed when assessing the internalization of CXCR3, which showed a decrease in the total number of receptors after 3 hours incubation with CXCL11. Internalization via β-arrestin recruitment and subsequent degradation has also been described for CXCR4, which also contains Ser/Thr clusters in its C-terminus [40]. On the other hand, we here show that once internalized, CXCR7 recycles back to the cell surface, which is in agreement with the absence of Ser/Thr clusters in its C-tail, corresponding to a more dynamic interaction with β-arrestin. By generating a chimera of CXCR7 with the C-terminus of CXCR3, we obtained a receptor unable to recycle and, most likely, subject to CXCR3-like mechanisms of receptor regulation. Unfortunately, we were unable to assess the regulation pattern of the reverse chimeric receptor corresponding to the CXCR3 with CXCR7 C-terminus due to the very limited cell surface expression of this mutant. However, our results suggest that the presence or absence of such clusters in CXCR7 C-tail determines the fate of receptors after endocytosis leading to downregulation or recycling, respectively. When monitoring the cell surface levels of CXCR7 after agonist exposure, it was observed that receptor internalization occurred in the first 30–60 minutes. Strikingly, recycling was observed in both conditions, upon chemokine removal (data not shown) but also when the incubation mixture was left for longer time periods. Recent publications by Naumann et al. and Luker et al. reconcile these events suggesting that CXCR7 mediates effective ligand internalization and targeting of the chemokine cargo for degradation. Such CXCR7-mediated depletion of CXCL12 [17], [29] would be sufficient to allow receptor recycling.

Apart from receptor phosphorylation, reversible ubiquitination constitutes a key regulatory mechanism for GPCRs [36]. This post-translational modification results in the covalent addition of the small protein ubiquitin to the intracellular lysine side chains of GPCRs with profound consequences for endocytic cycles of GPCRs [36]. In particular, CXCR4 has been shown to undergo CXCL12-induced ubiquitination resulting in lysosomal degradation of the receptor. CXCR4 ubiquitination occurs after receptor internalization and is mediated by the E3 ubiquitin ligase AIP4 via its interaction with β-arrestin [44], highlighting a novel function of β-arrestins in endosomal sorting of GPCRs. Deubiquitination of CXCR4, and therefore its escape from degradation, has been shown to be mediated by USP14 [45], while the deubiquitinating enzyme (DUB) USP8 has been shown to participate indirectly on CXCR4 regulation by modulating the dynamics of the signaling endosomes [46]. Similar to CXCR4, CXCR7 contains several intracellular lysines. Therefore, we hypothesized a potential role for ubiquitination in the regulation of CXCR7. By using two complementary techniques, i.e. co-immunoprecipitation and BRET^2^ [38], we show that in the basal state, CXCR7 is ubiquitinated while this is not the case for mutant receptors lacking the entire C-tail (CXCR7 ΔC) or the intracellular lysine residues (CXCR7 K/A). Furthermore, we prove that the Lys residues on the C-terminus of CXCR7 are responsible of receptor ubiquitination by showing that the chimeric CXCR3 receptor containing the CXCR7 C-terminus is ubiquitinated, while the WT CXCR3 and the CXCR7 receptor with the CXCR3 C-terminus are not. Moreover, we observed that receptor activation by CXCL12 results in reversible de-ubiquitination since subsequent removal of the chemokine from the media partially restored the ubiquitinated receptor levels detected in the basal state. These results are in contrast to what has been described for CXCR4, and highlight the differences that could potentially underlie distinct functions and/or patterns of expression of the two CXCL12 binding receptors.

**Figure 8:**
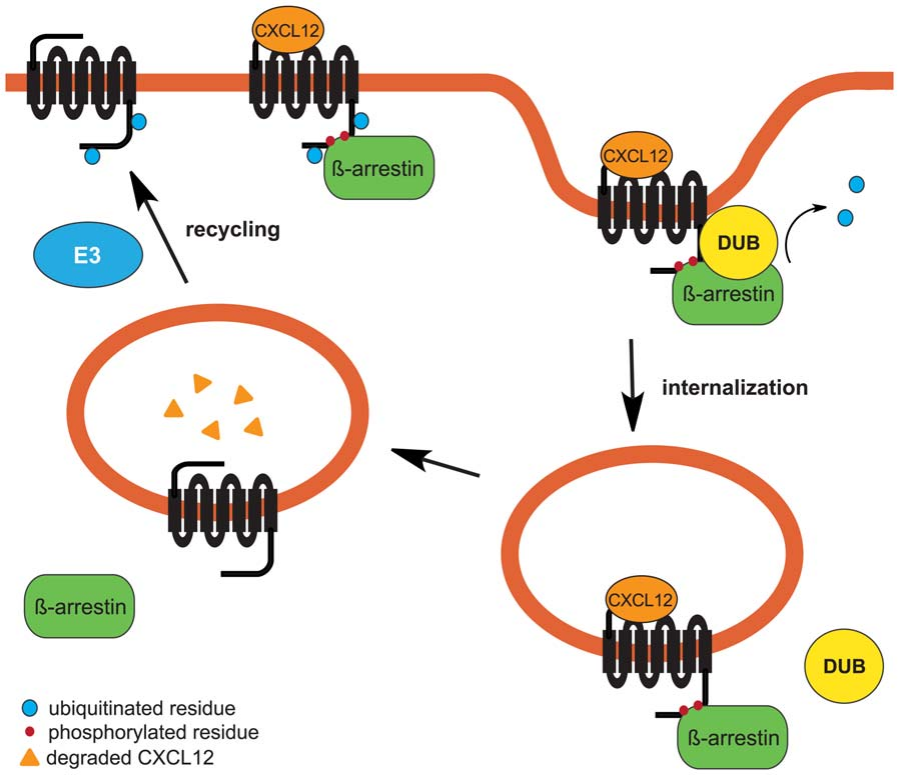
Proposed model for regulation of CXCR7 trafficking. CXCR7 requires ubiquitination of the Lys residues of its C-tail in order to reach the cell surface. Receptor activation by CXCL12 and subsequent phosphorylation of the C-terminal Ser/Thr residues results in β-arrestin recruitment by CXCR7 and receptor internalization in CCPs. In addition, β-arrestin scaffolds the interaction of CXCR7 with an unknown de-ubiquitinating enzyme (DUB) responsible for receptor deubiquitination. After chemokine degradation in early endosomes and due to the transient interaction of CXCR7 with β-arrestin, release of β-arrestin (and DUB) from the endocytosed receptor results in a CXCR7 able to undergo ubiquitination by a specific E3 ligase (E3) and subsequent delivery of the recycled receptor to the cell surface.

So far, the only receptor reported to undergo agonist-mediated de-ubiquitination is PAR-1, for which ubiquitination has been shown to play a role in regulation of receptor trafficking and a mutant PAR-1 lacking intracellular lysines has been shown to be constitutively internalized [47]. Our data suggests that we could propose a similar scenario for CXCR7 as we have observed that a CXCR7 receptor with mutated C-terminal lysines is unable to reach the cell surface, but is retained intracellularly and displays an increased basal interaction with β-arrestin2. Therefore, the observation of differential receptor trafficking of the different mutants has led us to propose a preliminary model based on ubiquitination as an essential determinant for CXCR7 regulation (**Fig. 8**). According to this model, CXCR7 ubiquitination is necessary for cell surface delivery of the receptor and the absence of this ubiquitination would lead to a constitutively internalized receptor. Upon CXCL12 stimulation, CXCR7 phosphorylation promotes β-arrestin recruitment. We hypothesize that β-arrestin would scaffold the interaction with a de-ubiquitinating enzyme (DUB) responsible for CXCR7 de-ubiquitination. The de-ubiquitinated receptor would subsequently be internalized. Due to the dynamic interaction of β-arrestin and the CXCR7 C-terminus, uncoupling of β-arrestin and interacting proteins would render the receptor able to undergo ubiquitination and recycle to the cell surface. According to this model, CXCR7 ΔC, despite not being ubiquitinated would be delivered at the cell surface due to the absence of the motifs responsible for β-arrestin interaction and endocytosis. Additionally, the absence of arrestin recruitment would prevent CXCR7 de-ubiquitination and therefore stabilize the receptor at the cell surface, as is observed for the CXCR7 ST/A mutant. As a consequence, the overall ubiquitination status of a cell would influence the regulation of CXCR7 and, therefore, its function. It will therefore be of key importance in the future to validate the previous findings in cells natively expressing CXCR7 and relate them to the ubiquitination machinery of such cells. Future studies will also focus on the identification of the E3 ligase and DUB interacting with CXCR7 and/or β-arrestin.

Recently, Sanchez-Alcaniz et al. reported that CXCR7 indirectly regulates the expression of CXCR4 in cortical interneurons. Such regulation is achieved by the dynamic internalization of CXCR7 and prevention of excessive CXCR4 desensitization and endocytosis [12]. CXCR7 would thus “fine-tune” the concentrations of CXCL12, thereby enabling directional migration of interneurons. Therefore, the differential ubiquitination patterns of these two receptors upon agonist stimulation could reflect a potential mechanism of achieving such dynamic regulation.

It is well established that deregulation of ubiquitin pathways [48] as well as defective endocytosis [49] result in the development of diseases, including many types of tumors. In this context, recent studies have shown that CXCR7 expression increases tumor formation and metastasis for some cancers [4], [7], which suggests that this receptor plays an important role in this process. However, the ubiquitination state of CXCR7 under these pathophysiological conditions remains to be explored. Recent reports also suggest that one important function of CXCR7 is to prevent degradation of CXCR4 [12]. Therefore, high expression of CXCR7 in tumor cells may contribute to excessive signaling through CXCR4, a landmark of the pathophysiology of WHIM syndrome, which is also associated with tumor growth and metastasis formation [50].

In summary, we have identified ubiquitination as a post-translational modification of CXCR7 responsible for its regulation and we have, for the first time, shown the constitutive ubiquitinated state of a chemokine receptor. Hence, future studies are essential to establish not only the role of ubiquitination processes in CXCR7-related cancer progression but also the potential of therapies targeting the blockade of CXCR7 in cells in which it is co-expressed with CXCR4.

## Materials and Methods

### Materials

All materials for tissue culture were purchased from PAA Laboratories GmbH (Paschen, Austria). Lipofectamine 2000 was purchased from Invitrogen (Paisley, UK). Poly-L-lysine, cycloheximide, bafilomycin A1, o-phenylenediamine and monoclonal anti-HA-Agarose conjugate were from Sigma-Aldrich (St. Louis, MO, USA), Coelenterazine-h was obtained from Promega (Madison, WI, USA). DeepBlueC (Coelenterazine 400a) was obtained from Biotium (Hayward, CA, USA). [^125^I]-CXCL12 (2200 Cimmol^−1^) and [^35^S]GTPγS were obtained from PerkinElmer Life and Analytical Sciences (Boston, MA, USA). Unlabeled chemokines were purchased from PeproTech (Rocky Hill, NJ, USA). Monoclonal antibody anti-CXCR7 (clone 11G8) and anti-CXCR3 (mAb160) were from R&D Systems. Monoclonal antibody anti-β-arrestin1/2 (clone D24H9) was purchased from Cell Signaling Technology (Boston, MA, USA). The CAMYEL biosensor was purchased from ATCC (#ATCC-MBA-277). β-arrestin1-YFP was a kind gift from C. Hoffmann, β-arrestin (319–418) from J. Benovic, HA-Ub from F. Mayor Jr., and Ub-GFP^2^ constructs were a generous gift from M. Bouvier. The CXCR7-RLuc construct was generated using PCR, by substituting the stopcodon of CXCR7 with a SpeI/NotI linker and fusing it in frame to *RLuc*, as described previously [51]. For siRNA transfection experiments, Dharmacon siRNA control pools (#D-001810-10) and siRNA pools targeting β-arrestin1 (#L-011971) and β-arrestin2 (#L-007292) were purchased from Thermo Scientific (Epsom, UK).

### Cell Culture and Transfection

HEK293T cells and HEK293 cells stably expressing human CXCR7 (HEK293/CXCR7), were grown at 37°C and 5% CO_2_ in Dulbecco's modified Eagle's medium (DMEM) supplemented with 10% FBS, penicillin, and streptomycin. HEK293T cells were transfected using linear polyethyleneimine (PEI) with a molecular weight of 25 kDa (Polysciences, Warrington, PA) as described previously [52]. In β-arrestin knockdown experiments, HEK293 cells stably expressing CXCR7 were transfected with a total of 250 pmol of siRNAs against both β-arrestin1 and -2 (1∶1), using lipofectamine 2000 according to standard protocol. The growth medium was replenished 5 hours after transfection. In any case, the day after transfection, cells were trypsinized, resuspended into culture medium, and plated in the corresponding poly-L-lysine-coated assay plates. Pertussis Toxin (PTX) treatment was performed overnight at a final concentration of 25 ng/ml.

### Membrane preparation and Chemokine Binding

Membrane preparation and competition bindings were performed as described previously [52]. Briefly, cell membrane fractions from HEK293T cells expressing CXCR7 were prepared by washing the cells twice with ice-cold PBS and centrifuging them at 1500 *g* for 10 min. The pellet was resuspended in ice-cold membrane buffer (15 mM Tris, pH 7.5, 1 mM EGTA, 0.3 mM EDTA, and 2 mM MgCl_2_), and homogenized using a Teflon-glass homogenizer and rotor. The membranes were subjected to two freeze-thaw cycles using liquid N_2_, and centrifuged at 40,000 *g* for 25 min. The pellet was resuspended in Tris-sucrose buffer (20 mM Tris, pH 7.4, and 250 mM Sucrose) and aliquots were frozen in liquid nitrogen. For [^125^I]-CXCL12 competition binding experiments, 1 µg/well of membranes were incubated in 96-well plates in binding buffer (50 mM HEPES, pH 7.4, 1 mM CaCl_2_, 5 mM MgCl_2_, 100 mM NaCl, and 0.5% (w/v) BSA) with approximately 70 pM [^125^I]-CXCL12 and various concentrations of displacer for two hours at room temperature. Membranes were harvested by filtration through Unifilter GF/C plates (Perkin-Elmer) presoaked with 0.5% PEI, using ice-cold wash buffer (50 mM HEPES, pH 7.4, 1 mM CaCl_2_, 5 mM MgCl_2_, and 500 mM NaCl). Radioactivity was measured using a MicroBeta scintillation counter (Perkin-Elmer).

### [^35^S]-GTPγS binding assay

5 µg/well of cell membranes were incubated with CXCL11 and CXCL12 in assay buffer (50 mM Hepes, 10 mM MgCl_2_, 100 mM NaCl, pH 7.2) supplemented with 3 µM GDP and 500 pM of [^35^S]-GTPγS. Incubations were placed at room temperature for 1 hour before harvesting the membranes by filtration through Unifilter GF/B plates. [^35^S]-GTPγS binding was determined using a Microbeta scintillation counter.

### cAMP biosensor BRET assay

The experimental procedure for this assay has been adapted from Masri et al. [53], using the CAMYEL BRET-based biosensor for cAMP. Twenty-four hours post-transfection, cells were trypsinized and seeded in poly-L-lysine coated white 96-well microplates. The cells were then cultured for an additional 24 h. Cells were rinsed once with Hank's Balanced Salt Solution (HBSS) to remove traces of phenol red and were then incubated in fresh HBSS. The *Renilla* luciferase (*RLuc*) substrate coelenterazine-h was added to reach a final concentration of 5 µM. The non-specific phosphodiesterase inhibitor IBMX was added simultaneously to a final concentration of 40 µM. For measuring effects of chemokines on cAMP levels, they were added 5 min after coelenterazine-h. Forskolin was added 5 min later, yielding a final concentration of 10 µM. After 5 min of incubation with forskolin the YFP emission (535 nm), as well as the RLuc emission (480 nm), were sequentially recorded for every assay point using a Victor^3^ multilabel counter (Perkin-Elmer). The BRET signal (BRET ratio) was determined by calculating the ratio of the light emitted at 505 to 555 nm (YFP) to the light emitted at 465 to 505 nm (RLuc).

### β-arrestin recruitment BRET

For β-arrestin recruitment experiments, HEK293T cells were transfected with a 1∶4 ratio of cDNA coding for CXCR7-RLuc and β-arrestin1- or -2-YFP (total DNA 2.5 µg per million cells). 24 hours post-transfection, cells were trypsinized and seeded in poly-L-lysine coated white 96-well culture plates (Greiner). The cells were then cultured for an additional 24 h. Cells were rinsed once with HBSS to remove traces of phenol red and were then incubated in fresh HBSS. The *Renilla* luciferase (*RLuc*) substrate coelenterazine-h was added to reach a final concentration of 5 µM. After 5 min of incubation with coelenterazine-h, the corresponding agonist was added, and incubated for 10 additional minutes. After 10 min readings were collected using a Victor^3^ instrument (PerkinElmer) and BRET ratios were calculated. The values were corrected by subtracting the background signal detected when the Receptor-*RLuc* construct was expressed alone. In inhibition experiments, cells where incubated with the anti-CXCR7 antibody 8F11 30 min prior to the addition of coelenterazine-h.

### Cell surface receptor expression and internalization ELISA

Transiently transfected HEK293T- or HEK293/CXCR7 cells, were trypsinized and replated in poly-L-Lysine coated 48-well plates. After 24 hours, cells were incubated with medium containing CXCL11, CXCL12 at 10^−8^ M or vehicle for several time periods in case of internalization experiments, or directly fixed when only receptor expression levels were determined. After ligand treatment, cells were subjected to three sequential acid washes (DMEM pH∼2), fully removing the chemokines from the receptors, such that they did not interfere with antibody binding (data not shown). Subsequently, cells were fixed with 4% formaldehyde in Tris-buffered saline (TBS). When stated, cells were permeabilized with TBS/0.5% NP-40. After blocking with 1% skim milk in 0.1 M NaHCO_3_ pH 8.6, cells were incubated overnight at 4°C with anti-CXCR7 or anti-CXCR3 antibodies (11G8 [54] and mAB160, respectively) in TBS (50 mM Tris, 150 mM NaCl, pH 7.5) containing 0.1% BSA. The cells were then washed three times with TBS, and incubated with goat anti-mouse HRP-conjugated secondary antibody (Bio-Rad). Subsequently, cells were incubated with substrate buffer containing 2 mM o-phenylenediamine, 35 mM citric acid, 66 mM Na_2_HPO_4_, and 0.015% H_2_O_2_ at pH 5.6. The coloring reaction was stopped by adding 1 M H_2_SO_4_, and the absorption at 490 nm was determined in a Powerwave X340 absorbance plate reader (BioTek).

### Immunofluorescence

HEK293 cells expressing CXCR7 growing on poly-L-lysine-coated coverslips were incubated with DMEM containing CXCL11, CXCL12 or vehicle for different time periods. Next, the cells were washed three times with acid wash (DMEM pH∼2), fixed with 4% formaldehyde in PBS and blocked with 3% skim milk in PBS or simultaneously permeabilized using 3% skim milk containing 0.15% Triton X-100/PBS. Then the cells were incubated consecutively with primary anti-CXCR7 11G8 monoclonal antibody and Alexa488-conjugated anti-mouse secondary antibody (Molecular Probes, Invitrogen). An Olympus FSX100 BioImaging Navigator was used for detection of fluorescence and the capturing of images.

### Whole cell binding

HEK293T cells expressing wt or mutated CXCR7 were plated 100,000 cells/well into a 48-well assay plate (Greiner). The next day, the medium was aspirated and the cells were incubated in binding buffer (50 mM Hepes pH 7.4, 1 mM CaCl_2_, 5 mM MgCl_2_ and 100 mM NaCl) containing ∼70 pM of [^125^I]-CXCL12 in the presence and absence of unlabeled ligands. After 4 hours at 4°C, the cells were washed three times with ice-cold wash buffer (50 mM Hepes pH 7.4, 1 mM CaCl_2_, 5 mM MgCl_2_ and 500 mM NaCl), lysed and bound radioactivity was measured in a Wallac Compugamma counter (PerkinElmer).

### Detection of receptor ubiquitination by immunoprecipitation

A total of five 10-cm plates of transfected HEK293T cells were used for every co-immunoprecipitation condition. 48 hours after transfection cells were rinsed twice and collected in a final volume of 5 ml of ice-cold PBS (1 ml per plate). Cells were pelleted by centrifugation for 3 min at 1000 rpm and subsequently lysed, homogenized and incubated at 4°C for 30 min with 1 ml of lysis buffer (1% NP-40, 1 mM EDTA, 150 mM NaCl, 10% Glycerol and 1 mM CaCl_2_). The lysates were then centrifuged at 14,000 rpm and the supernatant was recovered. 50 µl of these lysates were collected for later analysis and the remaining volume was incubated with agarose-conjugated HA-antibody for 90 min at 4°C on a rotating shaker. Immunoprecipitates were then washed three times by centrifugation with wash buffer (0.1% Triton X-100, 50 mM Tris pH 7.4, 300 mM NaCl, 5 mM EDTA) and an additional final wash with cold PBS. Finally, samples were eluted with sample buffer and processed for Western Blot analysis.

### SDS-PAGE and Western Blot

Cell lysates were subjected to SDS-PAGE analysis using 4–12% Bis-Tris gels (BioRad). After electrophoresis, proteins were transferred onto nitrocellulose membranes that were incubated in 5% non-fat milk and 0.1% Tween-20/TBS solution at room temperature on a rotating shaker for 2 h to block nonspecific binding sites. The membrane was incubated overnight with the corresponding antibody and detected using a horseradish peroxidase-linked secondary antibody. Immunoblots were developed by application of enhanced chemiluminescence solution (Pierce).

### BRET^2^ monitoring of receptor ubiquitination

To assess receptor ubiquitination using BRET^2^, HEK293T cells were co-transfected in a 1∶4 ratio of RLuc-tagged receptor (CXCR7 wt, CXCR7 ΔC, CXCR7 ST/A, CXCR4 or CXCR3) and GFP^2^-tagged Ubiquitin (wt or G75A, G76A mutant). 24 hours post-transfection, cells were trypsinized and seeded in poly-L-lysine coated white 96-well culture plates. The cells were then cultured for an additional 24 h. On the day of the experiment, cells were rinsed once with Hank's Balanced Salt Solution (HBSS) to remove traces of phenol red and were then incubated in fresh HBSS for additional 30 min. Subsequently, cells were incubated with 10^−8^ M of chemokine for 30 min. BRET^2^ measurements were collected 20 s after the addition of the *Renilla* luciferase (*RLuc*) substrate Coelenterazine 400a (Biotium), at a final concentration of 5 µM. Readings were collected with Victor^3^ instrument (PerkinElmer) detecting the signals in the 370–450 and 500–530 nm ranges. BRET^2^ ratios were calculated as described previously [30].*Data*

### Analysis

Nonlinear regression analysis of the data and calculation of affinity values was performed using Prism 5.04 (GraphPad Software Inc., San Diego, CA).

## Supporting Information

Figure S1 **CXCR7 does not activate Gα_i/o_ proteins.** (**A**) [^35^S]GTPγS binding assay in membranes of HEK293 cells transiently transfected with CXCR7 (triangles) or stably expressing CXCR3 (circles). Membranes were incubated with increasing concentrations of CXCL11 (black symbols) or CXCL12 (open symbols). Results are expressed as fold over basal [^35^S]GTPγS binding from three independent experiments and represent mean ± SEM. (**B**) Inhibition of forskolin-induced cAMP accumulation in HEK293 cells transiently transfected with CXCR7 or stably expressing CXCR3 and simultaneously transfected with the cAMP BRET biosensor CAMYEL. Data results from three independent experiments and is expressed as percentage of forskolin (Fsk) response and represent mean ± SEM.
(PDF)

Figure S2 **Cell surface expression of RLuc-tagged receptors.** **Surface** expression of RLuc -tagged CXCR7 constructs was assessed by [^125^I]CXCL12 whole cell binding. Data represent the mean ± SEM of 3 experiments each performed in triplicate.
(PDF)

Figure S3 **CXCR7 recycles after agonist stimulation while CXCR3 downregulates upon prolonged exposure to its ligand.** Receptor surface expression was assessed by ELISA in HEK293T cells transiently transfected with wt CXCR7 or wt CXCR3. To assess for total receptor expression cells were permeabilized after fixation with 0.5% NP-40. Data represent the mean ± SEM of 3 experiments each performed in triplicate.
(PDF)

Figure S4 **CXCR7 K/A colocalization with β-arrestin2.** HEK293T cells were transiently transfected with CXCR7 wt or K/A (red channel) and β-arrestin2-YFP (green channel). Cells were fixed and permeabilized prior to the immunodetection of CXCR7 with the 11G8 anti-CXCR7 antibody and an anti-mouse Alexa546-conjugated secondary antibody. Scale bar represents 10 µm.
(PDF)

Figure S5 **CXCR7 K/A shows increased basal interaction with β-arrestin2.** HEK293T cells coexpressing RLuc-tagged CXCR7 wt or K/A mutant and YFP-tagged β-arrestin2 were stimulated with 10^−8^ M of CXCL12 prior to BRET measurements. Results are expressed as fold of basal Net BRET as described in Materials and Methods. Data represent the mean ± SEM of 3 experiments each performed in triplicate.
(PDF)

Table S1 **Amino acid sequence of the mutated C-tails of CXCR7.** Bold letters indicate the introduced changes from the CXCR7 original sequence. The conserved NPXXY motif is underlined as a reference.
(DOC)

Table S2 **CXCL12 binding affinities for mutant CXCR7 receptors.** pK_d_ values were obtained by [^125^I]-CXCL12 homologous competition binding on membrane preparations of cells expressing CXCR7 WT or mutant receptors.
(DOC)
