# Supplementary figures and images for: Ubiquitination of CXCR7 Controls Receptor Trafficking
Source: PLoS One. 2012 Mar 23;7(3):e34192. doi: 10.1371/journal.pone.0034192 (PMC3311620; doi:10.1371/journal.pone.0034192)

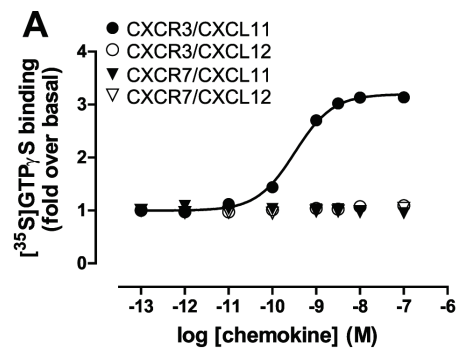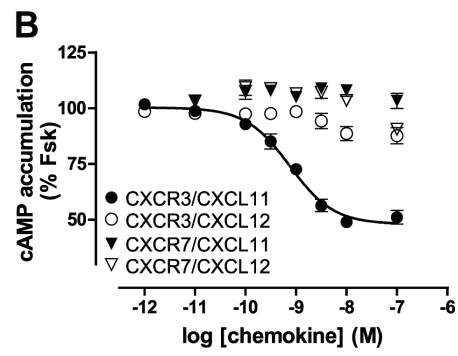

Supplement: Figure S1 — CXCR7 does not activate Gαi/o proteins. (A) [35S]GTPγS binding assay in membranes of HEK293 cells transiently transfected with CXCR7 (triangles) or stably expressing CXCR3 (circles). Membranes were incubated with increasing concentrations of CXCL11 (black symbols) or CXCL12 (open symbols). Results are expressed as fold over basal [35S]GTPγS binding from three independent experiments and represent mean ± SEM. (B) Inhibition of forskolin-induced cAMP accumulation in HEK293 cells transiently transfected with CXCR7 or stably expressing CXCR3 and simultaneously transfected with the cAMP BRET biosensor CAMYEL. Data results from three independent experiments and is expressed as percentage of forskolin (Fsk) response and represent mean ± SEM. (PDF) [file pone.0034192.s001.pdf]

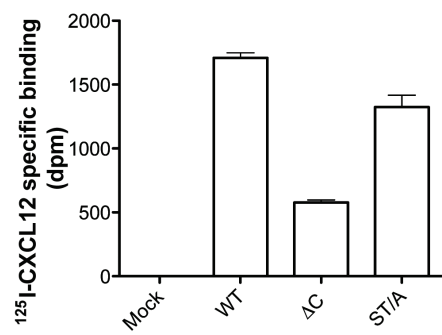

Supplement: Figure S2 — Cell surface expression of RLuc-tagged receptors. Surface expression of RLuc -tagged CXCR7 constructs was assessed by [125I]CXCL12 whole cell binding. Data represent the mean ± SEM of 3 experiments each performed in triplicate. (PDF) [file pone.0034192.s002.pdf]

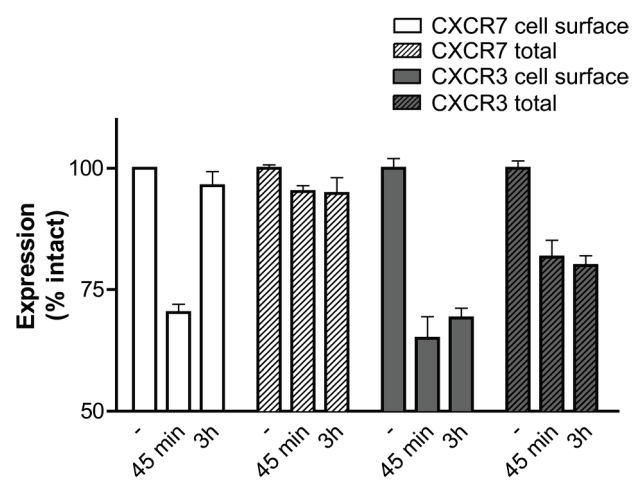

Supplement: Figure S3 — CXCR7 recycles after agonist stimulation while CXCR3 downregulates upon prolonged exposure to its ligand. Receptor surface expression was assessed by ELISA in HEK293T cells transiently transfected with wt CXCR7 or wt CXCR3. To assess for total receptor expression cells were permeabilized after fixation with 0.5% NP-40. Data represent the mean ± SEM of 3 experiments each performed in triplicate. (PDF) [file pone.0034192.s003.pdf]

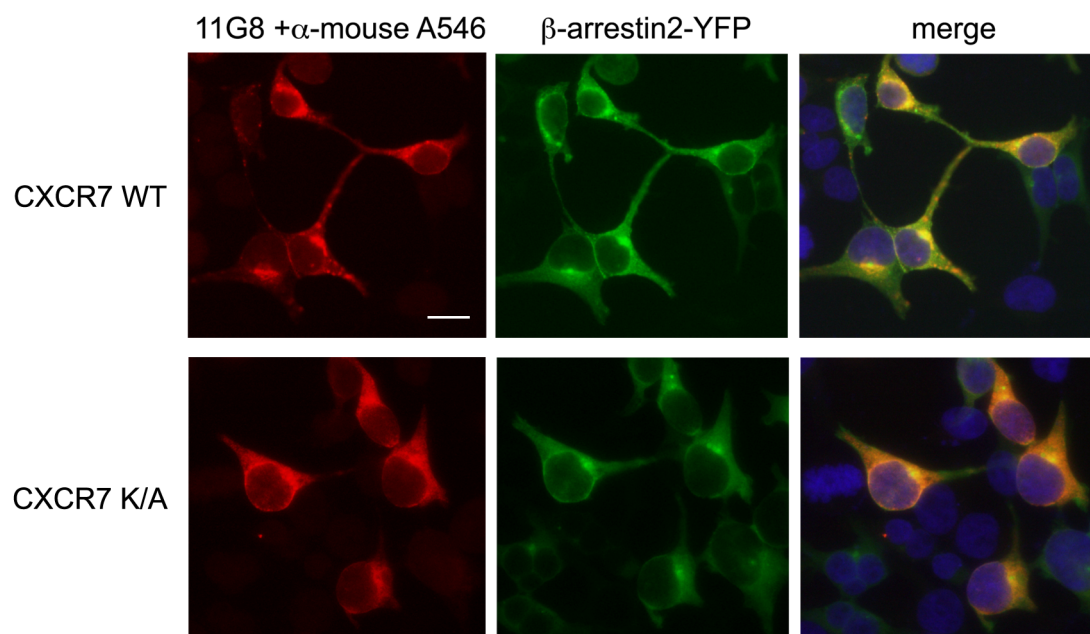

Supplement: Figure S4 — CXCR7 K/A colocalization with β-arrestin2. HEK293T cells were transiently transfected with CXCR7 wt or K/A (red channel) and β-arrestin2-YFP (green channel). Cells were fixed and permeabilized prior to the immunodetection of CXCR7 with the 11G8 anti-CXCR7 antibody and an anti-mouse Alexa546-conjugated secondary antibody. Scale bar represents 10 µm. (PDF) [file pone.0034192.s004.pdf]

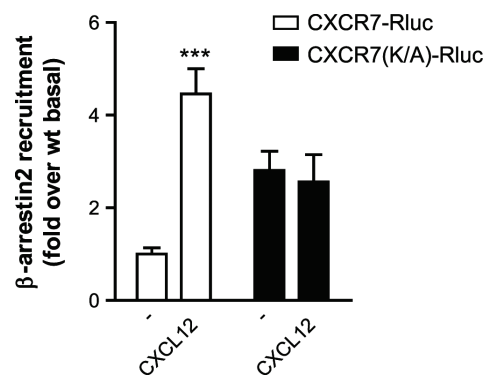

Supplement: Figure S5 — CXCR7 K/A shows increased basal interaction with β-arrestin2. HEK293T cells coexpressing RLuc-tagged CXCR7 wt or K/A mutant and YFP-tagged β-arrestin2 were stimulated with 10−8 M of CXCL12 prior to BRET measurements. Results are expressed as fold of basal Net BRET as described in Materials and Methods. Data represent the mean ± SEM of 3 experiments each performed in triplicate. (PDF) [file pone.0034192.s005.pdf]
