# Supplementary material for: Ubiquitination of CXCR7 Controls Receptor Trafficking
Source: PLoS One. 2012 Mar 23;7(3):e34192. doi: 10.1371/journal.pone.0034192 (PMC3311620; doi:10.1371/journal.pone.0034192)
Supplement: Table S1 — Amino acid sequence of the mutated C-tails of CXCR7. Bold letters indicate the introduced changes from the CXCR7 original sequence. The conserved NPXXY motif is underlined as a reference. (DOC) [file pone.0034192.s006.doc]

| **Construct** | **DNA sequence** |
| --- | --- |
| CXCR7 WT | NPVLYSFINRNYRYELMKAFIFKYSAKTGLTKLIDASRVSETEYSALEQSTK |
| CXCR7-X3 | NPVLYSFINRNYR**ERMWMLLLRLGCPNQRGLQRQPSSSRRDSSWSETSEASYSGL** |
| CXCR7 ΔC | NPVLYSFINRNYR |
| CXCR7 ST/A | NPVLYSFINRNYRYELMKAFIFKY**A**AK**A**GL**A**KLIDA**A**RV**A**E**A**EY**A**ALEQ**AA**K |
| CXCR7 K/A | NPVLYSFINRNYRYELM**A**AFIF**A**YSA**A**TGLT**A**LIDASRVSETEYSALEQST**A** |
