# Supplementary material for: Ubiquitination of CXCR7 Controls Receptor Trafficking
Source: PLoS One. 2012 Mar 23;7(3):e34192. doi: 10.1371/journal.pone.0034192 (PMC3311620; doi:10.1371/journal.pone.0034192)
Supplement: Table S2 — CXCL12 binding affinities for mutant CXCR7 receptors. pKd values were obtained by [125I]-CXCL12 homologous competition binding on membrane preparations of cells expressing CXCR7 WT or mutant receptors. (DOC) [file pone.0034192.s007.doc]

| **Construct** | **Affinity (pK_d_ ± SEM)** |
| --- | --- |
| CXCR7 WT | 9.7 ± 0.1 |
| CXCR7-X3 | 9.5 ± 0.1 |
| CXCR7 ΔC | 9.2 ± 0.1 |
| CXCR7 ST/A | 10.6 ± 0.2 |
| CXCR7 K/A | 9.5 ± 0.1 |
